# RNA-seq based detection of differentially expressed genes in the skeletal muscle of Duroc pigs with distinct lipid profiles

**DOI:** 10.1038/srep40005

**Published:** 2017-02-14

**Authors:** T. F. Cardoso, A. Cánovas, O. Canela-Xandri, R. González-Prendes, M. Amills, R. Quintanilla

**Affiliations:** 1 Department of Animal Genetics, Center for Research in Agricultural Genomics (CSIC-IRTA-UAB-UB), Universitat Autònoma de Barcelona, Bellaterra, 08193, Spain; 2 CAPES Foundation, Ministry of Education of Brazil, Brasilia D. F., Zip Code 70.040-020, Brazil; 3 Animal Breeding and Genetics Program, Institute for Research and Technology in Food and Agriculture (IRTA), Torre Marimon, Caldes de Montbui 08140, Spain; 4 Departament de Ciència Animal i dels Aliments, Universitat Autònoma de Barcelona, Bellaterra, 08193, Spain

## Abstract

We have used a RNA-seq approach to investigate differential expression in the skeletal muscle of swine (N = 52) with divergent lipid profiles *i.e.* HIGH (increased intramuscular fat and muscle saturated and monounsaturated fatty acid contents, higher serum lipid concentrations and fatness) and LOW pigs (leaner and with an increased muscle polyunsaturated fatty acid content). The number of mRNAs and non-coding RNAs (ncRNAs) expressed in the porcine *gluteus medius* muscle were 18,104 and 1,558, respectively. At the nominal level of significance (*P*-value ≤ 0.05), we detected 1,430 mRNA and 12 non-coding RNA (ncRNA) transcripts as differentially expressed (DE) in the *gluteus medius* muscle of HIGH *vs* LOW pigs. This smaller contribution of ncRNAs to differential expression may have biological and technical reasons. We performed a second analysis, that was more stringent (*P*-value ≤ 0.01 and fold-change ≥ 1.5), and only 96 and 0 mRNA-and ncRNA-encoding genes happened to be DE, respectively. The subset of DE mRNA genes was enriched in pathways related with lipid (lipogenesis and triacylglycerol degradation) and glucose metabolism. Moreover, HIGH pigs showed a more lipogenic profile than their LOW counterparts.

Several RNA-seq studies have been carried out on different pig breeds in order to identify genes involved in fat deposition and meat quality^1^,^2^. Besides analysing gene expression differences, these studies aimed to dissect the complex networks of pathways and genes that determine porcine phenotypes of economic interest. In this way, the expression patterns of porcine liver, *longissimus dorsi* and abdominal fat were examined in two full-sib hybrid pigs with extreme phenotypes for growth and fatness traits^3^. The proportion of tissue-specific mRNA transcripts happened to be quite modest (<10%) and several microRNAs (miRNAs) were differentially expressed (DE) across tissues. Other studies analysing differential gene expression in muscle, fat and liver tissues of Iberian x Landrace pigs with extreme phenotypes for muscle fatty acid (FA) composition revealed that DE loci are integrated in common pathways related with LXR/RXR activation, peroxisome proliferator-activated receptors (PPARs) and β-oxidation^1^,^4^,^5^. A recent analysis comparing Iberian and Iberian x Duroc pigs also identified LXR/RXR activation and cholesterol synthesis as enriched pathways in the set of DE genes^2^. In contrast, the potential role of ncRNAs in muscle fat deposition has been scarcely studied in pigs^4^,^6^.

In a previous experiment, we demonstrated that genes involved in FA uptake, lipogenesis, triacylglycerol synthesis, lipolysis and insulin signalling are DE in the skeletal muscle of Duroc pigs with divergent lipid phenotypes^7^. One drawback of this study was that gene expression was measured with microarrays, which have a limited dynamic range, sensitivity (specially for low-abundance transcripts) and specificity. Moreover, the expression of non-coding RNAs could not be measured with Affymetrix porcine microarrays. In the current work, we aimed to circumvent all these limitations by analysing, through a RNA-seq approach, the muscle transcriptome of a subset of these Duroc pigs. Our goal was to determine the relative contributions of protein-coding and non-coding RNAs to differential expression in the skeletal muscle of pigs with distinct lipid profiles.

## Results

The RNA-seq experiment allowed us generating an average of 133 million paired-end reads per sample and 72.8% of them were successfully mapped to the pig *Sscrofa10.2* genome assembly. The percentages of exonic and intronic reads were 91.4% and 8.6%, respectively. After quality control analysis, four samples were discarded. Thereby, we used a final dataset of 26 animals per group (HIGH and LOW) to identify DE genes.

### Differential expression of mRNA encoding genes

A total of 1,430 mRNA genes happened to be DE when considering exclusively a significance threshold of *P*-value ≤ 0.05 (Supplementary Table S1). Only 76 of these 1,430 mRNA-encoding genes were identified as DE by Cánovas *et al*.^7^ when they compared the gene expression of HIGH and LOW pigs retrieved from the same population employed by us (Supplementary Figure 1, Supplementary Table S2). When we performed a more stringent analysis (*P*-value ≤ 0.01 and fold-change ≥1.5), 96 genes were DE (Supplementary Table S3). Moreover, twenty-one genes remained significant after correction for multiple testing (*q*-value ≤ 0.05 and fold-change ≥1.5) as shown in Table 1.

**Figure 1:**
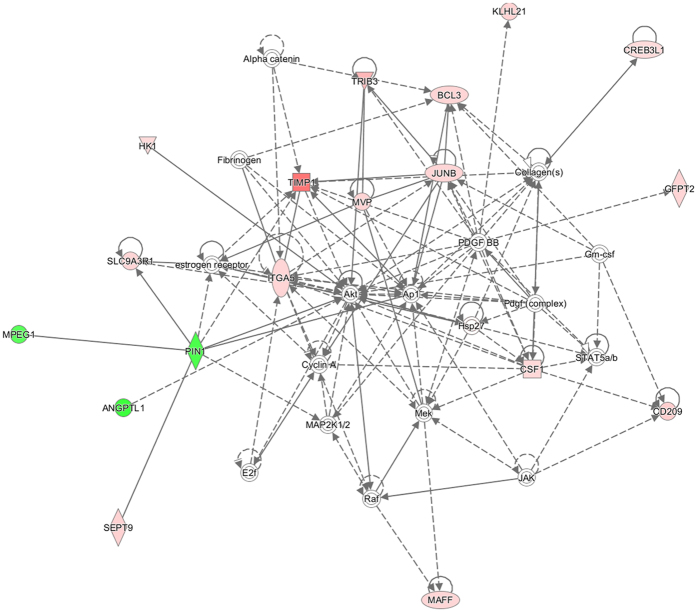
The top-scoring regulatory network identified with the IPA software corresponded to Cardiovascular Disease, Cardiovascular System Development and Function, Organismal Injury and Abnormalities. Genes that are upregulated and downregulated in HIGH pigs (when compared with the LOW ones) are displayed within red and green nodes, respectively. Solid and dashed lines between genes represent known direct and indirect gene interactions, respectively. The shapes of the nodes reflect the functional class of each gene product: transcriptional regulator (horizontal ellipse), transmembrane receptor (vertical ellipse), enzyme (vertical rhombus), cytokine/growth factor (square), kinase (inverted triangle) and complex/group/other (circle).

We used the IPA package (QIAGEN Redwood City, www.qiagen.com/ingenuity) to identify pathways to which DE genes belong to as well as to explore the existence of signalling networks connecting DE genes. Forty four pathways were significantly enriched in the dataset of 96 DE genes (Supplementary Table S4). This information should be interpreted with caution because, in general, pathways were represented by a small number of genes and statistical significance was not very high. Amongst the enriched pathways, it is worth to mention TR/RXR activation, synthesis of palmitate and stearate, FA biosynthesis, triacylglycerol degradation, and the conversion of acetate into acetyl-CoA (Table 2, Supplementary Table S4). A complementary analysis with the ReactomeFIViz app^8^ revealed 50 significant pathways (Supplementary Table S5). Differentially expressed mRNA genes were also grouped in gene regulatory networks with the IPA software. As shown in Supplementary Table S6, we found eleven regulatory networks related with a variety of functions, and the top-scoring one was that of *Cardiovascular Disease, Cardiovascular System Development and Function, Organismal Injury and Abnormalities* (Fig. 1 and Supplementary Table S6).

**Figure 2:**
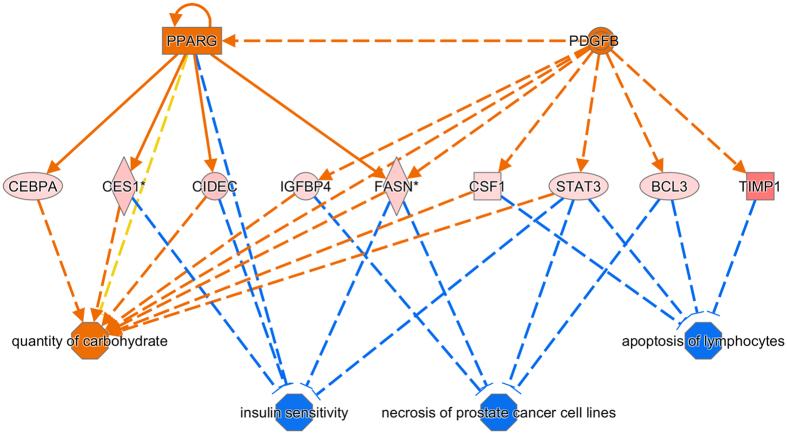
The Regulator Effects tool of the IPA package was employed to identify two major upstream regulators (*PPARG* and *PDGFB*) of the networks of differentially expressed genes. This tool integrates Upstream Regulator results with Downstream Effects results to build causal hypotheses that help to interpret what may be occurring upstream to cause particular phenotypic or functional outcomes downstream (http://www.ingenuity.com/products/ipa/ipa-spring-release-2014). In the upper tier, there are two upstream regulators (*PPARG* and *PDGFB*) predicted to be activated (orange color). In the middle tier, we can see the genes whose expression changes in response to the activation of upstream regulators (red = upregulation). The shapes of the nodes reflect the functional class of each gene product: enzyme (vertical rhombus), transcription regulator (vertical ellipse), cytokine/growth factor (square), ligand-dependent nuclear receptor (horizontal rectangle) and complex/group/other (circle). In the lower tier, the expected phenotypic consequences of changes in gene expression are shown by considering the Ingenuity Knowledge Base (absolute z-score > 2 and P-value < 0.05). The octagonal symbol defines Function, while solid and dashed lines between genes represent known direct and indirect gene interactions, respectively. Orange leads to activation, while blue leads to inhibition predicted relationships. Orange (predicted to be activated) and blue (predicted to be inhibited) lines represent relationships with causal consistency.

The Regulator Effects tool of the IPA package was employed to identify potential transcriptional regulators that may explain the differential patterns of expression observed between HIGH and LOW pigs (Fig. 2). By doing so, two main transcriptional regulators were identified *i.e.* peroxisome proliferator-activated receptor γ (*PPARG*) and platelet-derived growth factor BB (PDGFB). In the network shown in Fig. 2, these genes appear to be involved in an heterogeneous array of biological functions related with the quantity of carbohydrate, insulin sensitivity, necrosis of prostate cancer cell lines and apoptosis of lymphocytes. Indeed, the *PPARG* gene (*P*-value = 0.02 and FC = 1.36) is depicted as a key regulator of genes related with carbohydrate metabolism (*CEBPA, CES1, CIDEC*) and the inhibition of insulin sensitivity (*CES1, CIDEC, FASN*).

### Differential expression of non-coding RNAs

We identified 1,558 ncRNA transcripts expressed in the pig *gluteus medius* muscle, with sizes between 53 and 9,032 bp (Supplementary Table S7). Amongst these, 1,354 and 204 transcripts were classified as small (sncRNA) and long (lncRNA) non-coding RNAs, respectively. It is important to emphasize that the annotation of porcine ncRNAs is still very preliminar and it should be taken with caution. In general, sncRNA had orthologous sequences in other mammalian species, while lncRNAs were much less conserved (Table 3). We only detected 12 ncRNAs (11 lncRNAs and 1 sncRNA) that were DE at the nominal level (*P*-value ≤ 0.05), while none of these ncRNAs remained significant after correction for multiple testing (in all cases the *q*-value was non-significant, Table 4).

In addition, we identified 25 mRNA-encoding genes that mapped near (30 kb or less) to the subset of DE ncRNA loci (Table 5). This observation may have biological implications because ncRNAs often cis-regulate the expression of genes located in their vicinity. Within this list of neighbouring genes (Table 5), CU468594.8 (*P*-value =0.003 and FC =1.26) and MT-ND6 (*P*-value =0.038 and FC =−1.21) mRNAs are DE in HIGH *vs* LOW pigs (*P*-value < 0.05 and 1.2-fold change in expression).

## Discussion

### Divergent muscle mRNA expression profiles in pigs with extreme phenotypes for fatness traits

After correcting for multiple testing, twenty-one genes, displaying a wide array of functional roles, showed a significant DE between HIGH and LOW pigs (Table 1). For instance, *SLC27A4* is involved in the translocation of long-chain fatty acids across the plasma membrane^9^ while SFRP5 plays a role in anti-inflammatory and insulin-sensitizing processes^10^ and *AGO2* and *MVP* contribute to RNA interference^11^ and signal transduction and transport^12^, respectively. Two of the genes listed in Table 1 might be related with meat quality *i.e. RNF181*, which encodes a E3 ubiquitin-protein ligase that participates in the degradation of muscle proteins through the ubiquitin-proteasome system^13^, and *SDK1*, which has been associated with intramuscular fat (IMF) content in Large White pigs^14^.

The Spearman correlation between the microarray data reported by Canovas *et al*.^7^ in 68 HIGH and LOW pigs and RNA-seq data generated in the current study (N = 52) was 0.54. This value is comparable to what has been published in previous studies analysing gene expression in human brain cells (r = 0.61–0.67)^15^ and proliferating *vs* quiescent fibroblasts (r = 0.18–0.42)^16^. We also compared our dataset of DE genes with those detected by Canovas *et al*.^7^. As shown in Suppl Figure 1 the level of concordance was quite low (only 76 genes were simultaneously identified by both platforms). A modest overlap between microarray and RNA-seq data has been reported in previous studies. For instance, Trost *et al*.^16^ analysed the concordance between both types of data in fibroblasts cultured at two different developmental stages, and they just found an overlap of around 25% in the two lists of DE genes. This value is higher than the one reported by us, but it is important to highlight that the analysis of Trost *et al*.^16^ was based on a set of probes common to both platforms. Moreover, the microarray analysis performed by Canovas *et al*.^7^ was based on a dataset of around 68 pigs, while we used a subset of 52 individuals in our RNA-seq analysis. Trost *et al*.^16^ used quantitative real-time PCR as a third approach to validate microarray and RNA-seq data and they found that RNA-seq outperforms the microarray technology. However, differences between both methods are not dramatic *i.e.* the Spearman correlations between microarray and RNA-seq data *vs* qPCR validation results were 0.44 and 0.56, respectively. This means that both technologies detect different sets of DE expressed genes and, in consequence, they are complementary^17^. According to Wang *et al*.^18^, the magnitude of the treatment effect has a strong impact on the level of concordance between microarray and RNA-seq platforms *i.e.* large discrepancies can be anticipated when two similar biological conditions are compared. Low-abundance transcripts are another source of discrepancy between both methodological approaches^18^.

We found some evidence that pathways related with lipid synthesis (stearate, palmitate and FA synthesis) and catabolism (triacylglycerol degradation), glucose metabolism (glucose synthesis and degradation) and hormonal response (growth hormone signalling) were enriched in the set of DE genes (Table 2 and Supplementary Table S4). Similar results were obtained by Cánovas *et al*.^7^ *i.e*. they detected an overexpression of pathways related with the synthesis of FA and insulin signaling in HIGH pigs. Puig-Oliveras *et al*.^1^ compared the muscle mRNA expression of pigs with high saturated (SFA) and monounsaturated (MUFA) FA muscle contents against those with a high polyunsaturated FA (PUFA) content and also observed an enrichment of pathways related with fat deposition (PPAR and insulin signalling) in the set of DE genes. Insulin stimulates the absorption of glucose, which is a lipogenic substrate, and *PPARG* enhances triglyceride storage^19^. By using the same animal material employed by Puig-Oliveras *et al*.^1^, Corominas *et al*.^5^ observed an overexpression of genes belonging to the LXR/RXR activation pathway in the adipose tissue of pigs with high muscle SFA and MUFA contents. These results, which agree well with ours (Supplementary Table S4), make sense because liver X receptors are sterol-activated transcription factors that enhance lipogenesis^20^.

Though not all studies comparing pigs with divergent lipid phenotypes identify the same sets of pathways, an outcome that partly depends on the software and databases used as well as on the targeted tissue and phenotype variability, the general trend that emerges is that biochemical routes that promote lipid deposition are overexpressed in the skeletal muscle of fat pigs with high muscle SFA and MUFA contents. In close concordance with a previous study^7^, we have also found that one gene that promotes the catabolism of triglycerides, carboxylesterase 1 (*CES1*), is strongly upregulated in HIGH pigs (*P*-value = 0.0006, FC = 2.4). The CES1 protein has hydrolase activity and its inactivation leads to hyperlipidemia and increased fat deposition in peripheral tissues, obesity, fatty liver, hyperinsulinemia and insulin insensitivity and a decreased energy expenditure^21^. According to Cánovas and coworkers^7^, the upregulation of lipolytic genes in HIGH pigs suggests the existence of a cycle where triacylglycerols are continuously synthesized and degraded. However, we have also detected the downregulation of lipolytic genes such as lipase C, hepatic type (LIPC, *P*-value = 0.002, FC = −1.5)^22^, a feature that suggests that the mechanisms that promote an adequate balance between anabolic and catabolic lipid metabolism routes are highly complex.

Analysis of the data with the IPA software (QIAGEN) showed that the top-scoring regulatory network was *Cardiovascular Disease, Cardiovascular System Development and Function, Organismal Injury and Abnormalities*, a result that it is not surprising given the tight relationship between lipoprotein metabolism and cardiovascular risk^23^. In the network shown in Fig. 1, the V-Akt murine thymoma viral oncogene homolog molecule (AKT) occupies a central position, having connections with several DE lipid-related genes (*e.g., TRIB3, TIMP1 and ITGA5*). Interestingly, AKT is one of the main regulators of glucose homeostasis^24^, a feature that is consistent with the existence of tight links between lipid and carbohydrate metabolism.

When we used the Regulator Effects tool of IPA, the *PPARG* and *PDGFB* genes were predicted to be major transcriptional regulators of the set of 96 DE loci (Fig. 2). The PPARG transcription factor is critically required for adipogenesis, being a powerful modulator of whole-body lipid homeostasis and insulin sensitivity^25^. Polymorphism in the *PPARG* gene is associated with individual susceptibility to type 2 diabetes, obesity and body mass index^26^. In our study, *PPARG* is upregulated (*P*-value = 0.02 and FC = 1.36) in HIGH pigs and appears to regulate several genes, such as *CEBPA (P*-value = 0.009 and FC = 1.64), *CES1 (P*-value = 0.0004 and FC = 2.03), *CIDEC (P*-value = 0.0005 and FC = 2.46) and *FASN (P*-value = 0.0009 and FC = 2), that play distinct roles in lipid metabolism (http://www.genome.jp/kegg/pathway.html).

### Limited contribution of the non-coding RNA transcriptome to differential expression between HIGH and LOW pigs

Non-coding RNAs have been shown to regulate gene expression by interacting with chromatin complexes, working as RNA enhancers, recruiting or assembling certain proteins and interacting with other RNAs at the post-transcriptional level^27^. In consequence they may play a fundamental role in the metabolism of the porcine skeletal muscle. In our study, we have identified 1,558 muscle-expressed ncRNA transcripts (Supplementary Table S7). The total number of ncRNAs in the pig genome is currently unknown, but Zhou *et al*.^28^ highlighted the existence of at least 6,621 long intergenic non-coding RNAs (lincRNA) transcripts encoded by 4,515 gene loci. In humans, 58,648 lncRNA encoding loci have been identified so far^29^. In our dataset (Table 3), the degree of evolutionary conservation of sncRNAs happened to be much higher than that of lncRNAS. Zhou *et al*.^28^ characterized the porcine lincRNA transcriptome and found that only 40% of the transcripts had a detectable human lincRNA ortholog. This scarcity of orthologous sequences can be due, in part, to the poor annotation of ncRNAs in all investigated species.

There is growing evidence that there might be a positive correlation between the expression of ncRNAs and nearby mRNA encoding genes, suggesting that the former may regulate the expression of the latter^30^. We investigated this issue by analysing if there are DE protein-coding genes in the vicinity of any of the 12 DE ncRNAs identified in our work (*P*-value ≤ 0.05, Tables 4 and 5). Two protein-coding genes, *i.e.* mitochondrially encoded NADH:ubiquinone oxidoreductase core subunit 6 (*MT-ND6*) and CU468594.8, fulfilled this condition (*P*-value ≤ 0.05 and FC ≥ 1.2, Table 5). The *MT-ND6* gene encodes a NADH dehydrogenase that catalyses the oxidation of NADH by ubiquinone, an essential step in the mitochondrial electron transport chain^31^. The *CU468594.8* locus is orthologous to human solute carrier family 52-riboflavin transporter, member 2 (*SLC52A2*). Riboflavin is the precursor of flavin adenine dinucleotide (FAD) and flavin mononucleotide (FMN), two essential cofactors that participate in a wide range of redox reactions^32^,^33^.

We aimed to ascertain if differences amongst HIGH and LOW pigs, in terms of IMF content and composition, are mainly due to the DE of either mRNA or ncRNA encoding genes. When considering a nominal *P*-value of 0.05 as a threshold of significance, the number of DE ncRNAs (12 loci) was much smaller than that of DE mRNAs (1,430 loci), even if we take into account that the number of expressed mRNAs (18,104) was also higher than that of ncRNAs (1,558). Moreover, none of the DE ncRNAs remained significant after correction for multiple testing. In a recent experiment, the transcriptome of pig endometrial samples collected at different pregnancy stages was characterized, and 2,376 transcripts were identified as DE in pairwise comparisons^34^. Only 12% of these transcripts corresponded to lncRNAs indicating that changes in the endometrial transcriptome associated with pregnancy mainly affect the expression of protein-coding genes. However, studies performed in humans indicate a much more balanced contribution of mRNAs and ncRNAs to differential expression. For instance, Wang *et al*.^35^ investigated the expression patterns of peripheral leukocytes of healthy and autistic individuals and identified 3,929 and 2,591 DE lncRNAs and mRNAs, respectively. Similarly, Zhou *et al*.^36^ identified 891 and 576 DE mRNAs and lncRNAS, respectively, when comparing the expression patterns of ectopic and eutopic endometrial tissue. These differences between humans and pigs are probably the consequence of technical rather than biological causes, evidencing the pressing need of improving the genomic and functional annotation of porcine ncRNAs.

## Conclusions

By comparing the mRNA expression of HIGH and LOW pigs by RNA-seq, we have identified 96 loci displaying differential expression (*P*-value ≤ 0.01 and FC ≥ 1.5). Many of these loci were not detected in a previous microarray-based experiment, suggesting that distinct platforms detect different sets of DE genes. Lipid biosynthetic pathways were enriched in DE genes and upregulated in HIGH pigs, a result that is consistent with previous reports. We have also undertaken the analysis of non-coding RNAs, a feature that has been neglected in previous studies investigating the differential expression of porcine genes. Our results indicate that the number of DE non-coding RNAs is much lower than that of mRNAs, an outcome that might be partly explained by the poor annotation of porcine ncRNAs.

## Material and Methods

### Ethics statement

All experiments were performed in accordance with the ARRIVE guidelines (https://www.nc3rs.org.uk/arrive-guidelines). Animal care and management procedures were approved by the Ethical Committee of the Institut de Recerca i Tecnologia Agroalimentàries, IRTA.

### Animal Material

One population of 350 Duroc barrows belonging to 5 half-sib families, and distributed in 4 fattening batches was generated in 2003. All animals were kept under the same feeding and management conditions^37^. A wide array of growth, fatness, feed efficiency and carcass and meat quality traits were recorded in these animals, including weight, daily food intake, fat deposition, and IMF content and composition (C:12-C:22 interval) of the *gluteus medius* muscle^7^. By using a principal component analysis based on 13 lipid-related traits, we selected two groups of pigs, *i.e*. HIGH and LOW, displaying distinct phenotypic profiles^7^ (Supplementary Table S8). Compared with their LOW counterparts, HIGH pigs were fatter and they had a higher IMF, SFA and MUFA muscle contents as well as elevated serum lipid concentrations^7^. LOW pigs, in contrast, had a higher muscle PUFA content^7^.

### RNA isolation and library construction and sequencing

Total RNA was isolated from 56 porcine *gluteus medius* muscle samples (28 HIGH and 28 LOW) by using the acid phenol method implemented in the RiboPure kit (Ambion, Austin, TX). Total RNA was quantified in a Nanodrop ND-1000 spectrophotometer, checked for purity and integrity in a Bioanalyzer-2100 device (Agilent Technologies, Inc., Santa Clara, CA) and submitted to the Centre Nacional d’Anàlisi Genòmica (CNAG, http://www.cnag.cat) for sequencing. Libraries were prepared using the TruSeq RNA Sample Preparation Kit (Illumina Inc) according to the protocols recommended by the manufacturer. Each library was paired-end sequenced (2 × 75 bp) by using the TruSeq SBS Kit v3-HS, in a HiSeq2000 platform.

### Bioinformatic analyses

All bioinformatic analyses were performed with the CLC Bio Workbench software (CLC Bio, Aarhus, Denmark). Quality control was carried out with the NGS Core Tools, considering several parameters based on the FastQC-project (http://www.bioinformatics.babraham.ac.uk/projects/fastqc/). We carried out per-sequence and per-base analyses to filter reads according to the following criteria: sequence-read distribution = 75 bp, 100% coverage in all bases, GC-content ~50%, ~25% of A, T, G and C nucleotide contributions, ambiguous base-content <0.1% and a Phred score higher than 30 (*i.e.* base-calling accuracy larger than 99.9%). Short sequence reads were assembled, mapped and annotated by using as template the pig reference genome version 10.2 (Sscrofa10.2- http://www.ensembl.org/info/data/ ftp/index.html). For mapping purposes, we just considered alignments with a length fraction of 0.7 and a similarity fraction of 0.8. Besides, two mismatches and three insertions and deletions per read were allowed.

Gene expression data were normalized by calculating the reads per kilobase per million mapped reads (RPKM)^38^. Using scales of abundance estimates by exon length and millions of mapped reads, original expression values were transformed and normalized. More specifically, data were transformed on a decimal logarithmic scale and a scaling algorithm was utilized for the normalization of average scores^39^. For the statistical analysis of differential expression, we used a two-tailed t-test that assumes a Gaussian distribution and homogeneous variances. This statistical test compares the mean expression levels in the two experimental groups (HIGH *vs* LOW) and evaluates the significance of the difference relative to the variance of the data within the groups. Multiple testing correction was performed by using a false-discovery rate approach (cut-off = 0.05) implemented in the QVALUE R package^40^. Fold-Change was computed as the ratio of HIGH *vs* LOW gene expressions (a negative FC means that the affected gene is upregulated in LOW pigs).

Ingenuity Pathway Analysis (IPA, QIAGEN Redwood City, www.qiagen.com/ingenuity) was used to identify gene ontologies, pathways, and regulatory networks to which DE genes belong to, as well as upstream regulators. Ingenuity Pathway Analysis can transform a set of genes into a number of relevant networks based on comprehensive records maintained in the Ingenuity Pathways Knowledge Base. Networks are presented as graphs depicting the biological relationships between genes/gene products. Genes are shown as nodes, and the molecular relationship between two nodes is represented with either a solid (direct interactions) or a dashed (indirect interactions) line. The analysis of upstream regulators considers every possible transcription factor and upstream regulator contained in the Ingenuity Knowledge Base repository as well as their predicted effects on gene expression (inferred from the scientific literature). Then, this tool analyses if the patterns of expression observed in the DE genes can be explained by the activation/inhibition of any of these regulators through the calculation of a z-score *i.e.* a statistical measure of the match between expected relationship direction between the regulator and its targets and observed gene expression^41^. A parallel analysis was performed with the Cytoscape software^42^ by using the ReactomeFIViz app^8^. IPA and Cytoscape analyses were performed on a subset of DE genes, with *P*-value ≤ 0.01 and a FC ≥ 1.5. Transcript classification and the search of homologs of porcine ncRNAs in other mammalian species were carried out with tools implemented in the BioMart web interface (http://www.ensembl.org/biomart/martview).

## Additional Information

**How to cite this article:** Cardoso, T.F. *et al*. RNA-seq based detection of differentially expressed genes in the skeletal muscle of Duroc pigs with distinct lipid profiles. *Sci. Rep.* **7**, 40005; doi: 10.1038/srep40005 (2017).

**Publisher's note:** Springer Nature remains neutral with regard to jurisdictional claims in published maps and institutional affiliations.

## Supplementary Material

Supplementary Information

## Figures and Tables

**Table 1:** List of the most significant differentially expressed genes in HIGH and LOW pigs after correcting for multiple testing (*q*-value ≤ 0.05 and fold-change ≥ 1.5).

Ensembl ID	Gene name	Fold-Change	*P*-value	*q*-value
ENSSSCG00000005648	*SLC27A4*	1.66	1.32E-06	4.28E-03
ENSSSCG00000027946	*MVP*	1.78	2.63E-06	5.97E-03
ENSSSCG00000017232	*SLC9A3R1*	1.72	1.26E-05	1.36E-02
ENSSSCG00000005935	*AGO2*	1.59	1.77E-05	1.43E-02
ENSSSCG00000003379	*KLHL21*	1.79	1.61E-05	1.43E-02
ENSSSCG00000011740	*SERPINI1*	−1.81	2.48E-05	1.72E-02
ENSSSCG00000001931	*GRAMD2*	−1.58	2.74E-05	1.76E-02
ENSSSCG00000011444	*NT5DC2*	1.54	3.26E-05	1.76E-02
ENSSSCG00000007574	*SDK1*	1.58	2.97E-05	1.76E-02
ENSSSCG00000007745	*SUMF2*	−1.54	4.21E-05	1.95E-02
ENSSSCG00000000293	*ITGA5*	1.72	4.46E-05	1.96E-02
ENSSSCG00000007133	*ACSS1*	1.51	5.18E-05	2.09E-02
ENSSSCG00000028814	*SOD3*	1.97	5.37E-05	2.09E-02
ENSSSCG00000006277	*SPIDR*	2.04	5.90E-05	2.19E-02
ENSSSCG00000007554	*ZFAND2A*	2.54	1.01E-04	2.88E-02
ENSSSCG00000003105	*SLC1A5*	1.67	1.17E-04	3.07E-02
ENSSSCG00000010529	*SFRP5*	2.03	1.31E-04	3.11E-02
ENSSSCG00000006245	*SDR16C5*	3.02	1.36E-04	3.15E-02
ENSSSCG00000013579	*CD209*	1.95	1.50E-04	3.31E-02
ENSSSCG00000008232	*RNF181*	−2.09	2.05E-04	3.57E-02
ENSSSCG00000030165	*MAFF*	1.67	2.22E-04	3.72E-02

A negative FC means that the affected gene is overexpressed in LOW pigs.

**Table 2:** IPA-based pathway analysis of the list of genes that are differentially expressed in HIGH and LOW pigs (*P*-value ≤ 0.01 and fold-change ≥ 1.5).

Ingenuity Canonical Pathways	−*log(p-value)*	Ratio	Nodes
Acute Myeloid Leukemia Signaling	3.22	4/91	*CEBPA, FLT3, RUNX1, STAT3*
Hematopoiesis from Pluripotent Stem Cells	2.98	3/47	*CD3E, CD8E, CSF1*
Primary Immunodeficiency Signaling	2.96	3/48	*CD3E, CD8E, ZAP70*
Hepatic Fibrosis/Hepatic Stellate Cell Activation	2.12	4/183	*CCR5, CSF1, IGFBP4, TIMP1*
TR/RXR Activation	2.08	3/98	*BCL3, FASN, SYT2*
Palmitate Biosynthesis I (Animals)	2.07	1/2	*FASN*
Fatty Acid Biosynthesis Initiation II	2.07	1/2	*FASN*
CTLA4 Signaling in Cytotoxic T Lymphocytes	2.07	3/99	*CD3E, CD8A, ZAP70*
Retinoate Biosynthesis I	2.04	2/34	*RDH5, SDR16C5*
Stearate Biosynthesis I (Animals)	2.02	2/35	*FASN, SLC27A4*

Ratio: number of DE genes in a pathway divided by the number of genes comprised in the same pathway.

**Table 3:** Evolutionary conservation of non-coding RNAs transcribed in the porcine *gluteus medius* muscle.

Transcript	Transcript Type	Number	Conserved ncRNA
**Small ncRNA**	miRNA	433	137
	misc_RNA	95	82
	Mt-rRNA	2	0
	Mt-tRNA	22	0
	rRNA	57	52
	snoRNA	417	395
	snRNA	328	273
**Long ncRNA**	Non coding	4	0
	Processed transcript	143	0
	Antisense	15	0
	lincRNA	42	0

miRNA = microRNAs; misc_RNA = miscellaneous other RNA; Mt-rRNA = Mitochondrial ribosomal RNA; Mt-tRNA = transfer RNA located in the mitochondrial genome; rRNA = ribosomal RNA; snoRNA = small nucleolar RNA; snRNA = small nuclear RNA; lincRNA = Long intergenic non-coding RNAs.

**Table 4:** List of non-coding RNAs that are differentially expressed (at the nominal level, *P*-value ≤ 0.05) in the *gluteus medius* muscle of HIGH and LOW pigs.

Ensembl ID	Gene ID	Size (bp)	Fold Change	*P*-value	Type of ncRNA
ENSSSCG00000031004	*CH242-227G20.3*	1833	−1.44	0.002	lincRNA
ENSSSCG00000031028	*CH242-15C8.2*	1495	−1.34	0.014	lincRNA
ENSSSCG00000015579	*PTGS2*	3601	−1.47	0.016	Processed transcript
ENSSSCG00000030904	*CU468594.10*	1083	−1.49	0.025	Non coding
ENSSSCG00000001227	*TMP-SLA-3*	1767	−1.31	0.026	Processed transcript
ENSSSCG00000030767	*TMP-SLA-5*	1147	−1.29	0.027	Processed transcript
ENSSSCG00000015549	*RNASEL*	2716	−1.87	0.028	Processed transcript
ENSSSCG00000018090	*Unavailable*	70	−2.05	0.036	Mt-tRNA
ENSSSCG00000001397	*TMP-CH242-74M17.4*	1726	−1.27	0.038	Processed transcript
ENSSSCG00000001227	*TMP-SLA-3*	1700	−1.3	0.043	Processed transcript
ENSSSCG00000004334	*MAP3K7-001*	2818	−1.72	0.044	Processed transcript
ENSSSCG00000015897	*IFIH1*	3720	−1.6	0.046	Processed transcript

A negative FC means that the affected gene is overexpressed in LOW pigs; lincRNA = Long intergenic non-coding RNAs, Mt-tRNA = transfer RNA located in the mitochondrial genome.

**Table 5:** Protein-encoding genes that map near (30 kb) to the subset of 12 differentially expressed ncRNAs (HIGH *vs* LOW pigs).

Non-coding RNA	Neighboring mRNA gene	Fold Change	*P*-value	RPKM-means LOW	RPKM-means HIGH
*CH242-15C8.2*	*USP9X*	−1.02	0.500	10.70	10.50
*CH242-227G20.3*	*PDK3*	−1.03	0.435	8.97	8.69
	*PCYT1B*	−1.07	0.461	0.35	0.33
***CU468594.10***	***CU468594.8***	**1.26**	**0.003**	1.40	1.77
	*CPSF1*	1.10	0.020	12.60	13.90
	*SLC39A4*	1.24	0.316	0.09	0.11
	*FBXL6*	−1.03	0.777	1.88	1.84
	*ADCK5*	−1.04	0.865	2.82	2.72
	*TMEM249*	1.07	0.587	0.08	0.08
**ENSSSCG00000018090**	*MT-ND2*	−1.15	0.024	5019.58	4372.26
	*MT-ATP6*	−1.13	0.033	25335.60	22504.43
	***MT-ND6***	**−1.21**	**0.038**	5199.05	4287.84
	*MT-COX2*	−1.12	0.051	20299.35	18062.05
	*MT-ND5*	−1.16	0.051	3264.12	2809.24
	*MT-COX1*	−1.13	0.064	24826.52	21886.25
	*MT-ND3*	−1.10	0.086	4413.22	4010.71
	*MT-CYTB*	−1.10	0.123	10033.49	9149.34
	*MT-ATP8*	−1.07	0.162	6421.54	5974.98
	*MT-COX3*	−1.08	0.164	31328.03	28896.14
	*MT-ND1*	−1.06	0.197	8412.34	7957.73
	*MT-ND4*	−1.04	0.243	5784.35	5564.63
	*MT-ND4L*	−1.03	0.289	2042.94	1980.50
*IFIH1*	*FAP*	−1.10	0.665	2.56	2.33
*RNASEL*	*RGS8*	2.22	0.300	0.11	0.24
*TMP-SLA-5* and *TMP-CH242-74M17.4*	*SLA-1*	−1.17	0.123	79.31	67.50

Differentially expressed ncRNAs and mRNAs (HIGH *vs* LOW pigs) *P*-value ≤ 0.05, Fold Change ≥ 1.2) are shown in bold. A negative Fold Change means that the affected gene is overexpressed in LOW pigs.
